# IL33 Is a Stomach Alarmin That Initiates a Skewed Th2 Response to Injury and Infection

**DOI:** 10.1016/j.jcmgh.2014.12.003

**Published:** 2015-01-03

**Authors:** Jon N. Buzzelli, Heather V. Chalinor, Daniel I. Pavlic, Philip Sutton, Trevelyan R. Menheniott, Andrew S. Giraud, Louise M. Judd

**Affiliations:** 1 Murdoch Children's Research Institute, Royal Children’s Hospital, Parkville, Victoria, Australia; 2 Department of Paediatrics, Royal Children’s Hospital, University of Melbourne, Parkville, Victoria, Australia; 3 Centre for Animal Biotechnology, School of Veterinary Science, University of Melbourne, Parkville, Victoria, Australia

**Keywords:** IL33, *Helicobacter pylori*, Inflammatory Response, Gastric Cancer, AB, Alcian blue, DC, dendritic cell, ELISA, enzyme-linked immunosorbent assay, ERK, extracellular signal–regulated kinase, FBS, fetal bovine serum, HBSS, Hank’s balanced salt solution, IL, interleukin, ILC, innate lymphoid cell, mRNA, messenger RNA, NF-κB, nuclear factor-κB, PAS, periodic acid–Schiff, PCR, polymerase chain reaction, QRT-PCR, quantitative reverse-transcription polymerase chain reaction, SMC, surface mucus cells, SPF, specific pathogen free, SS1, Sydney strain 1, STAT, signal transducer and activator of transcription, TFF, trefoil factor, Th, T-helper, WT, wild type

## Abstract

**Background & Aims:**

Interleukin (IL)33 is a recently described alarmin that is highly expressed in the gastric mucosa and potently activates Th2 immunity. It may play a pivotal role during *Helicobacter pylori* infection. Here, we delineate the role of IL33 in the normal gastric mucosa and in response to gastropathy.

**Methods:**

IL33 expression was evaluated in mice and human biopsy specimens infected with *H pylori* and in mice after dosing with aspirin. IL33 expression was localized in the gastric mucosa using immunofluorescence. Mice were given 1 or 7 daily doses of recombinant IL33 (1 μg/dose), and the stomach and the spleen responses were quantified morphologically, by flow cytometry and using quantitative reverse-transcription polymerase chain reaction and immunoblotting.

**Results:**

In mice, the IL33 protein was localized to the nucleus of a subpopulation of surface mucus cells, and co-localized with the surface mucus cell markers Ulex Europaeus 1 (UEA1), and Mucin 5AC (Muc5AC). A small proportion of IL33-positive epithelial cells also were Ki-67 positive. IL33 and its receptor Interleukin 1 receptor-like 1 (ST2) were increased 4-fold after acute (1-day) *H pylori* infection, however, this increase was not apparent after 7 days and IL33 expression was reduced 2-fold after 2 months. Similarly, human biopsy specimens positive for *H pylori* had a reduced IL33 expression. Chronic IL33 treatment in mice caused systemic activation of innate lymphoid cell 2 and polarization of macrophages to the M2 phenotype. In the stomach, IL33-treated mice developed transmural inflammation and mucous metaplasia that was mediated by Th2/signal transducer and activator of transcription 3 signaling. Rag-1^-/-^ mice, lacking mature lymphocytes, were protected from IL33-induced gastric pathology.

**Conclusions:**

IL33 is highly expressed in the gastric mucosa and promotes the activation of T helper 2–cytokine–expressing cells. The loss of IL33 expression after prolonged *H pylori* infection may be permissive for the T helper 1–biased immune response observed during *H pylori* infection and subsequent precancerous progression.

**Summary**

Interleukin 33 is a stomach alarmin that is increased immediately after gastric insult and infection but is suppressed during long-term *Helicobacter pylori* infection. Interleukin 33 potently activates gastric T helper 2 immunity, which suggests that its loss during *H pylori* infection may be important in establishing T helper 1 immunity.

Recently, it was suggested that a class of specialized immune regulators, called *alarmins*, are involved in activating an acute immune response after infection or injury.^1^ Alarmins describe a class of multifunctional cytokines released by necrotic cells in response to infection or injury to promote an innate and adaptive immune response.^1^ One such cytokine, interleukin 33 (IL33),^2^ enhances expression of T helper (Th)2 cytokines^3^ and activates multiple immune regulatory cells including group 2 innate lymphoid cells (ILC2),^4^ basophils,^5^, ^6^, ^7^ mast cells,^8^ eosinophils,^7^ natural killer T cells,^6^ and Th2 lymphocytes.^7^

Current research on IL33 has focused mainly on its role in lung pathology. However, IL33 also has been shown to provide protection during gastrointestinal infection and dextran sodium sulfate–induced colitis by vigorously enhancing Th2 immunity.^9^, ^10^, ^11^, ^12^ Collectively, these findings suggest that IL33 may be a crucial mediator of the immune response after damage or infection in epithelial tissues. IL33 is highly expressed in the stomach,^3^ however, little is known of its gastric function. In this study, we address which cells of the gastric mucosa express IL33, how IL33 expression changes with damage and infection, and characterize the function of IL33 in the stomach.

Similar to the lung and colon, the gastric mucosa is vulnerable to chronic infection and inflammation, which may promote serious pathologic outcomes such as peptic ulceration, intestinal metaplasia, and adenocarcinoma. The primary causative agent in this regard is *Helicobacter pylori*. The capacity of *H pylori* to colonize the gastric mucosa and promote a favorable environment for gastric disease is highly dependent on the response of the host immunity to insult and invasion. IL33 is highly expressed in the gastric epithelium^3^ and therefore may be an important factor in limiting *H pylori* colonization and consequent inflammatory pathology. Despite its disposition, a functional relationship between IL33 expression, *H pylori* infection, and resultant pathology has yet to be described. Here, we address the role of IL33 during the gastric immune response to the ulcerogen aspirin and in acute and chronic *H pylori* infection. We show IL33 protein to be localized predominately to the nuclei of a subset of surface mucus cells (SMCs), and that systemic administration of IL33 causes a Th2-biased immune response in the stomach and atypical gastric pathology. Furthermore, IL33 messenger RNA (mRNA) is lost in human gastric samples positive for *H pylori* and mice with prolonged *H pylori* infection; events that may result in the skewed Th1/Th17 immunity observed during *H pylori* infection and subsequent pathology.

## Materials and Methods

### Cells

MKN28 cells were grown in RPMI media containing 10% fetal bovine serum (FBS), 100 mmol/L nonessential amino acids (Sigma, St. Louis, MO), and 100 mmol/L penicillin-streptomycin (Sigma). One hour before experiments cell cultures were given fresh RPMI media containing 0.5% FBS.

#### Time course

Cells were given fresh media containing 100 ng/mL of recombinant human IL33 (Shenandoah, St. Louis, MO). Media was left on cells for 0, 1, 5, 15, 30, or 60 minutes before cells were collected (n = 3/time point). The 0-minute time point did not receive fresh media.

#### Dose response

Cells were given fresh media containing 0, 0.01, 0.1, 1, 10, or 100 ng/mL of recombinant human IL33 (Shenandoah) (n = 3/concentration). Media was left on cells for 5 minutes before cell harvest.

#### Cell harvesting

All cells were harvested using RIPA buffer containing 2 nmol/L of sodium fluoride, 2 nmol/L sodium orthovanadate, and 1 protease inhibitor cocktail per 10 mL of RIPA buffer solution (Roche Diagnostic, Indianapolis, IN).

### Mice

Wild-type (WT) mice were from a C57Bl/6 background, 10–12 weeks old. Most mice were housed in individually ventilated, high-efficiency particulate absorption–filtered cages (specific pathogen free [SPF] conditions), and a small subset of mice were kept in a different facility with covered, but not individually ventilated, shoe box cages (conventional conditions). All mice had autoclaved water and irradiated food. Genetically modified mice were genotyped by multiplex polymerase chain reaction (PCR) as previously described.^13^ Approval was obtained from Murdoch Children’s Research Institute.

### Aspirin Treatment

WT mice (n ≥ 5) and trefoil factor (TFF)2^-/-^ mice (n ≥ 4) were treated with 300 mg (200 μL) of aspirin (Sigma) via an oral gavage. Aspirin was suspended in 1% methylcellulose aqueous solution (Sigma) and control mice were gavaged with 200 μL of 1% methylcellulose. Mice were starved overnight before aspirin administration. One hour after treatment, mice were given food and killed either 4 or 24 hours after treatment.

### Cytokine Treatment

WT mice (n ≥ 5) were injected intraperitoneally once daily with 1 μg of recombinant human IL33 (Shenandoah) or saline for 7 days. One hour before culling, mice were given an additional dose of IL33.

### Tissue Preparation

Mouse stomachs were prepared and analyzed as previously described.^14^ Half of the fundus and antrum were collected in liquid nitrogen for RNA and protein. For histologic examination, bisected tissue was fixed in 4% paraformaldehyde in phosphate-buffered saline. The spleen also was collected for analysis. One half was frozen in liquid nitrogen for RNA and protein. The second half was fixed in 4% paraformaldehyde in phosphate-buffered saline.

### Cell Isolation and Flow Cytometry (Fluorescence-Activated Cell Sorting) Analysis

#### Spleen cell isolation

One third of the spleen was made into a single-cell suspension. Red blood cells were lysed with ammonium-Tris chloride buffer for 5 minutes at room temperature. Cell suspensions were stained with fluorescently labeled antibodies (Supplementary Table 1) in Hank’s balanced salt solution (HBSS) (2 mmol/L EDTA, 2% FBS), washed, and resuspended in HBSS for fluorescence-activated cell sorter analysis (LSRII and FACSDiva v6.1.1; Becton Dickinson, Franklin Lakes, NJ).

#### Stomach cell isolation

Stomachs were collected in HBSS (2 mmol/L EDTA, 2% FBS), perfused with digestion media (1 × HBSS [without calcium and magnesium], 5 mmol/L EDTA, 5% FBS, and 1 mmol/L dithiothreitol), and incubated at 37°C for 15 minutes. Stomachs then were cut and incubated in digestion media for a further 15 minutes, and then passed through a 70-μm cell strainer. Cell suspensions were centrifuged and resuspended in 1 × HBSS. Cells were analyzed as described for spleen samples.

#### Fluorescence-activated cell sorter analysis

Dead, autofluorescent, and aggregated cells were removed from the anlysis on the basis of forward scatter (FSC), side scatter (SSC), and propidium iodide staining. The total number of events for each cell type (Supplementary Table 1) was quantified using CountBright beads (#36950; Invitrogen, Carlsbad, CA).

### Macrophage Isolation and Analysis

To harvest peritoneal macrophages, 5 mL of HBSS (Sigma) containing 10 U/mL of heparin (Sigma) was injected into the peritoneal cavity (n = 5/group). Extracted cells were harvested, washed, resuspended in complete RPMI, and then plated onto 6-well culture plates. Plates were incubated at 37°C for 10 minutes to allow macrophages to adhere. Cultures then were washed to remove nonmacrophage cells. Macrophages were incubated overnight at 37°C. The next day, macrophage cultures were treated with either saline or 100 ng IL33/mL in fresh media. Cells were harvested using TRIzol reagent (Life Technologies, Carlsbad, CA).

### Collection of Human Biopsy Specimens

Biopsy specimens were obtained with written consent from the patients. Subjects’ histology and consent were taken and exclusions were made as follows: use of potentially damaging medications 4 weeks before biopsies, mucosal injury or tumor, other comorbidities affecting the gastric mucosa, or use of anticoagulants.^15^ Studies were approved by the Melbourne Health Organisation (#097.1998).

### Quantitative Reverse-Transcription PCR

Total RNA was harvested using TRIzol reagent (Life Technologies). RNA (3 μg) (n ≥ 5 animals/group) was reverse-transcribed into complementary DNA using Moloney murine leukemia virus reverse transcriptase (Promega, Madison, WI) primed with oligo(dT). Quantitative reverse transcription PCR (QRT-PCR) primers were designed using Primer Express (Applied Biosystems, Foster City, CA) (Supplementary Table 2). SYBR green chemistry was used with *rL32* as the internal reference gene. QRT-PCR conditions were as follows: 95°C for 10 minutes, 40 cycles of 95°C for 15 seconds, and 60°C for 15 seconds (AB7500; Applied Biosystems). Results were analyzed using sequence detector software, and relative fold differences were determined using the ΔΔCt method.

### Immunoblotting

Proteins (n ≥ 5 animals/group) were prepared with TRIzol (Life Technologies) and 20 μg of extract subjected to sodium dodecyl sulfate–polyacrylamide gel electrophoresis. Membranes were incubated with antibodies specific for signal transducer and activator of transcription (STAT)3, p-STAT3, extracellular signal–regulated kinase (ERK)1/2, p-ERK1/2, nuclear factor-κB (NF-κB), glyceraldehyde-3-phosphate dehydrogenase, β-actin (Supplementary Table 3) (Abcam, Cambridge, UK), or peroxide-conjugated secondary antibody, and visualized by enhanced chemiluminescence (Amersham, Buckinghamshire, UK). Quantification was performed using Quantity 1 software (Bio-Rad Laboratories, Hercules, CA), and phosphorylated:total protein ratios were determined from duplicate membranes.

### Immunohistochemistry

Paraffin sections (4 μm) on 3-aminopropyltriethoxysilane slides were subject to immunohistochemistry according to Supplementary Table 3. Antigen retrieval was performed with 10 mmol/L citric acid at 100°C for 30 minutes, followed by 30 minutes of cooling. Staining was completed with biotinylated secondary antibodies, avidin, and biotinylated horseradish-peroxidase complex (Vector Laboratories, Burlingame, CA), and 3,3’-diaminobenzidine and hematoxylin counterstained. For all staining reactions a control was performed with secondary antibody alone. For immunofluorescent staining, sections were prepared as described and staining was completed with fluorescent-conjugated secondary antibodies and cover slipped with Prolong Gold antifade-4′,6-diamidino-2-phenylindole mounting reagent (Invitrogen #P-36931). Representative images from each treatment group are shown.

### IL33 Enzyme-Linked Immunosorbent Assay

IL33 protein from mouse serum and stomach digests were analyzed using IL33 enzyme-linked immunosorbent assays (ELISAs) (eBioscience, San Diego, CA). For serum, 50 μL was used directly, whereas stomach digests were first analyzed by a Lowry assay and 30 μg of protein was diluted into 50 μL. The procedure was performed according to the manufacturer’s instructions.

### Quantitative Morphometry

All quantitative morphometry was performed by a blinded observer. Representative photos (n > 6) of each animal (n > 5) of histochemically or immunohistochemically stained sections were captured using a Coolpix 4500 digital camera (Nikon Instruments, Melville, NY) attached to a light microscope. Lengths of relevant cells were traced manually on these images using ImageJ software for Windows v1.38 (http://rsb.info.nih.gov/ij/index.html; National Institutes of Health, Bethesda, MD) to generate measurements. Measurements were converted to millimeters after comparison with a calibrated graticule.

### Statistical Analysis

All data were expressed as means ± SEM and statistical analysis was performed using 1-way analysis of variance and the appropriate parametric or nonparametric statistical test using Sigmastat (Jandel Scientific, San Rafael, CA). *P* values ≤ .05 were considered statistically significant.

## Results

### IL33 Expression Is Restricted to the Central Nervous System and Epithelial Tissues

**Figure 1:**
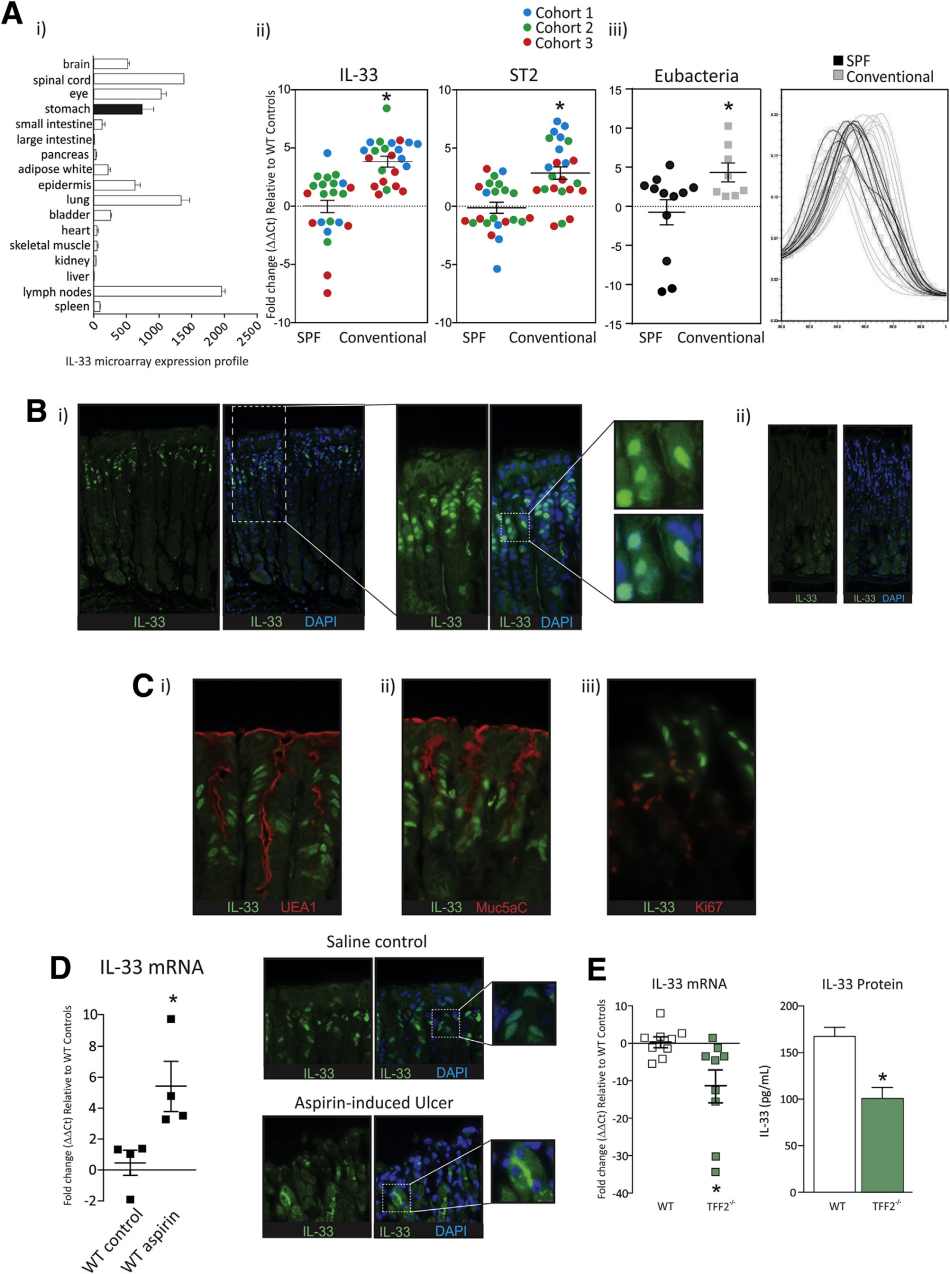
**IL33 localization in the gastric mucosa and mRNA expression increase owing to bacterial load and gastric insult.** (*Ai*) Comparison of IL33 expression in mouse tissues (repeated twice). (*Aii*) IL33 and ST2 mRNA expression in the fundus of mice housed in either SPF or conventional facilities as well as mouse gastric bacterial load (N ≥ 20) (repeated 3 times). (*Aiii*) Eubacterial 16S expression in the SPF and conventional animal facility. (*Bi*) IL33 protein localization in the gastric mucosa; (*ii*) IL33 immunofluorescence on the gastric mucosa of IL33^-/-^ mice (N = 5) (repeated twice). (*C*) Co-localization of IL33 with (*i*) UEA1, (*ii*) Muc5AC, and (*iii*) Ki-67 in the gastric mucosa (N = 6). (*D*) IL33 mRNA 4 hours after aspirin insult and IL33 protein localization at gastric ulcers (repeated twice, N = 6). (*E*) IL33 mRNA and protein levels in 12-week-old WT and TFF2^-/-^ mice measured by QRT-PCR and ELISA assay, respectively (N = 8) (repeated twice). ∗Statistically significant, *P* ≤ .05. DAPI, 4′,6-diamidino-2-phenylindole.

The BioGPS mouse microarray database was accessed to compare the tissue distribution of IL33 expression (http://biogps.org/#goto=genereport&id=77125) (Figure 1*Ai*). The outcome of 2 independent microarray experiments showed moderate to high expression of IL33 in the stomach, brain, spinal cord, eye, epidermis, lung, and lymph nodes, and low or no expression in the small and large intestines, pancreas, bladder, kidney, heart, liver, and spleen (Figure 1*Ai*). Therefore, the highest IL33 expression is found in epithelial tissues and the central nervous system. Interestingly, IL33 expression is not readily detectable in the large intestine, an organ containing high bacterial load and, similar to the stomach, susceptible to infectious disease. The expression profile of IL33 suggests that it may be critical in regulating gastric homeostasis and preventing bacterial overgrowth in the stomach, however, IL33 does not perform these functions uniformly in the gastrointestinal tract.

### Gastric IL33 mRNA Expression Is Dependent on Bacterial Load

IL33 has been shown to reduce colonization and pathologic outcomes in murine models of intestinal infection,^11^, ^12^, ^16^ raising the prospect that it is a gut alarmin. Because the disposition and function of IL33 in the stomach is unknown, we initially investigated whether gastric bacterial load influenced IL33 expression. To do so, gastric IL33 expression, as well its receptor, ST2, was compared in mice housed in either SPF micro-isolator or conventional (filter top) housing. Mice used in these experiments were born and reared in their respective housing facility, and to ensure a robust finding mice were compared from 3 cohorts that were collected independently over the course of 12 months. Gastric IL33 and ST2 mRNA expression were increased in the conventional facility compared with the SPF facility (Figure 1*Aii*). A eubacteria QRT-PCR on the 16S RNA transcript showed that the stomachs of mice derived from the conventional housing facility had an increased and more diverse bacterial load compared with those from the SPF facility (Figure 1*Aiii*). These data suggest that, in the stomach, IL33 expression is increased with bacterial load and diversity, consistent with an alarmin function for this cytokine.

### IL33 Protein Is Localized to Surface Mucus Cells

Previous studies have shown that IL33 is detected in the gastric epithelium.^3^, ^17^ Immunofluorescence showed that IL33 is localized to the nucleus in a large subset of SMCs, in particular those at the base of the gastric pits, rather than directly in contact with the stomach lumen (Figure 1*Bi*). The nuclear localization was confirmed using 4′,6-diamidino-2-phenylindole counterstain (Figure 1*Bi*). IL33^-/-^ mice were used as controls, and no staining was observed in these mice (Figure 1*Bii*). To additionally characterize IL33 localization to SMCs, IL33 immunofluorescence was performed in conjunction with the SMC markers UEA1 and Muc5AC (Figure 1*Ci* and *ii*). IL33 protein expression was co-localized with both SMC markers, confirming our previous findings (Figure 1*Ci* and *ii*). SMCs are in direct contact with the stomach lumen and *H pylori* preferentially localizes to the gastric pits, suggesting that IL33 is placed ideally to mount an effective immune response after infection or injury.

Considering that IL33 resides within the nuclei of gastric pit cells and is not directly in contact with the stomach lumen we wanted to determine whether IL33 was expressed by gastric pit cells, presurface mucous cells, or progenitor cells. To address this we co-localized IL33 with Ki-67 (Figure 1*Ciii*). Staining showed Ki-67–positive cells were in close proximity with IL33-positive cells, and a small proportion of cells were double positive for Ki-67 and IL33, suggesting that IL33 is expressed by presurface mucous cells and gastric pit cells (Figure 1*Ciii*).

### IL33 Is Increased After Gastric Damage That Is Mediated Partially by TFF2

Alarmins are inflammatory mediators that respond rapidly after insult or injury. Therefore, to determine whether IL33 acts as a stomach alarmin, its expression and distribution was measured after gastric insult. Mice were given a single dose of aspirin (150 mg/kg in 1% methylcellulose) by oral gavage to produce erosive gastric injury,^18^ and were culled either 4 or 24 hours after treatment. Compared with WT controls, IL33 mRNA expression increased 4 hours after injury (Figure 1*D*), and returned to pre-injury levels within 24 hours (data not shown). In addition, at erosive sites, IL33 protein was released from the nucleus and localized to the cytoplasm in close proximity to the apical membrane of the cell (Figure 1*D*). This shows that IL33 responds immediately to gastric insult through re-localization and transcriptional changes and may be involved in gastric wound healing and restitution.

In the lung, the mucous cell homeostatic regulator TFF2 has been shown to drive IL33 expression.^12^ TFF2 is expressed most highly in the gastric mucosa, and loss of TFF2 promotes gastric pathology.^19^ To determine whether TFF2 also influences gastric IL33 expression, steady-state IL33 mRNA was measured in the gastric fundus of TFF2^-/-^ mice at 12 weeks. In TFF2^-/-^ mice, IL33 mRNA expression was decreased substantially compared with WT controls (Figure 1*E*). In addition, IL33 protein, quantified by ELISA assay, was reduced by approximately 40% in TFF2^-/-^ mice compared with WT mice (Figure 1*E*). These findings show that, in the stomach, IL33 expression is at least partly regulated by TFF2.

### IL33 Expression Is Increased in Response to Acute *H pylori* Infection, but Is Suppressed in Chronic Infection in Mice and Human Beings

**Figure 2:**
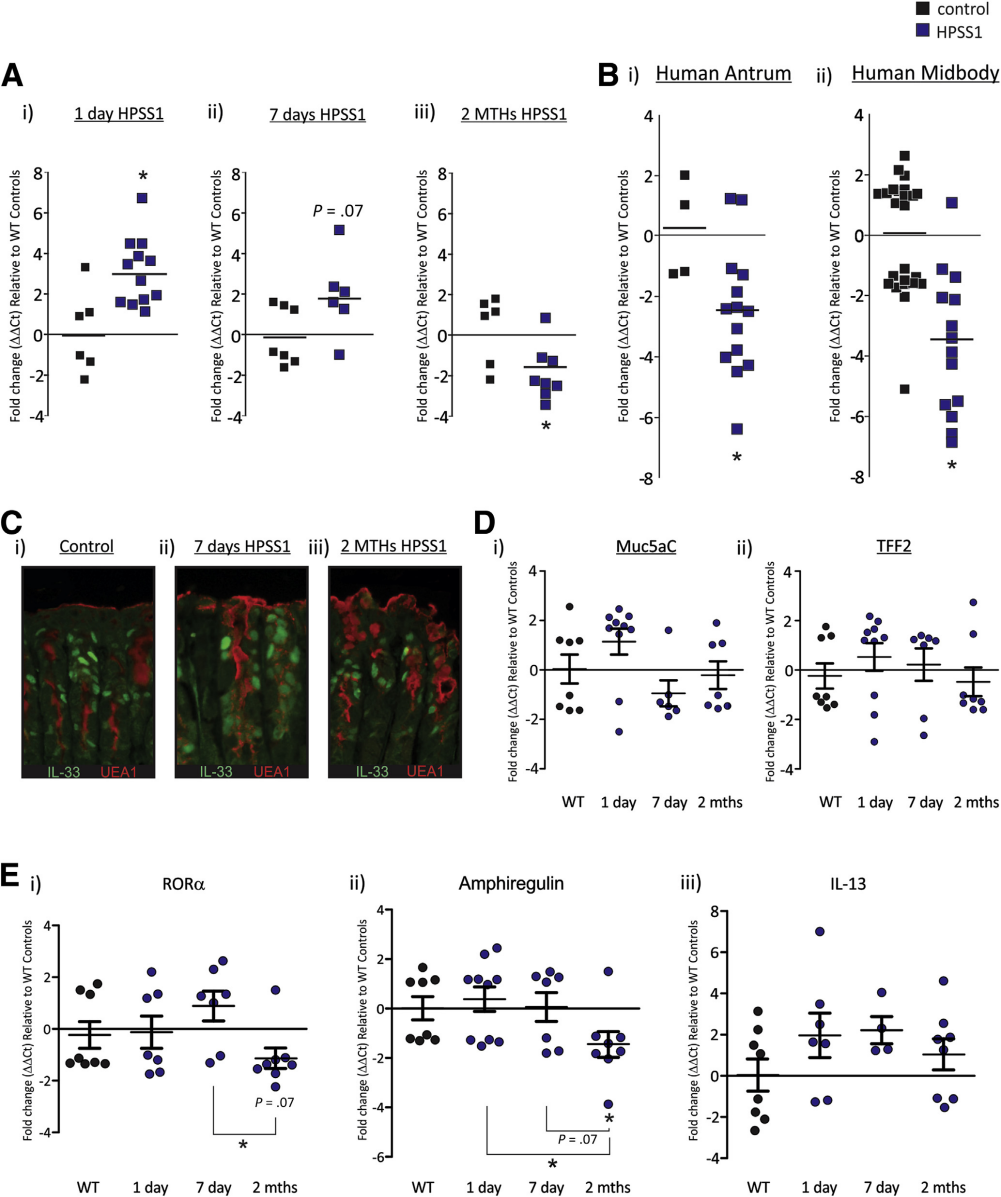
**IL33 mRNA expression in the stomach increases after acute *H pylori* infection and steadily decreases with more chronic infection.** (*A*) IL33 mRNA expression after *H pylori SS1* infection in mice; (*i*) 1 day, (*ii*) 7 days, and (*iii*) 2 months relative to uninfected mice and standardized to the housekeeper L32 (N ≥ 6) (repeated twice). (*B*) IL33 mRNA expression in human biopsy specimens positive for *H pylori* infection relative to uninfected biopsy specimens and standardized to the housekeeper L32; (*i*) antrum and (*ii*) midbody (N ≥ 12). (*C*) Co-localization of IL33 with UEA1 in *H pylori*–SS1–infected mice: (*i*) control, (*ii*) 7 days, and (*iii*) 2 months (N = 5). (*D*) mRNA expression of (*i*) Muc5AC and (*ii*) TFF2 after 1 day, 7 days, and 2 months of *H pylori*–SS1 infection, and standardized to the housekeeper L32 (N ≥ 6). (*E*) Expression of the ILC2 genes: (*i*) RORα, (*ii*) amphiregulin, and (*iii*) IL13 after 1 day, 7 days, and 2 months of *H pylori*–SS1 infection, and standardized to the housekeeper L32 (N ≥ 6). ∗Statistically significant, *P* ≤ .05.

To determine the consequences of acute and chronic *H pylori* infection on IL33 expression, mice were infected with *H pylori*–Sydney strain 1 (SS1) for 1 day, 7 days, or 2 months, and IL33 mRNA was quantified by QRT-PCR. After 1 day of infection, IL33 mRNA expression was increased relative to uninfected controls (Figure 2*Ai*). IL33 mRNA expression still was increased 7 days after *H pylori*–SS1 infection, however, this increase was less than that observed in the 1-day cohort, and was not significantly different from controls (Figure 2*Aii*). Two months of *H pylori*–SS1 infection resulted in a significant reduction in IL33 mRNA expression (Figure 2*Aiii*). To extend our understanding of the interplay between IL33 expression and *H pylori* infection we collected human gastric biopsy specimens from individuals chronically infected with *H pylori* and uninfected controls. IL33 mRNA expression was decreased in the *H pylori*–positive antrum and midbody when compared with control biopsy specimens (Figure 2*B*). These data show that IL33 mRNA expression is induced rapidly after acute *H pylori* infection, and then suppressed with prolonged infection. Furthermore, we have shown that the effect of chronic *H pylori* infection on IL33 mRNA expression is similar in human beings and mice. These data are consistent with an alarmin function for IL33 in the stomach.

**Figure 3:**
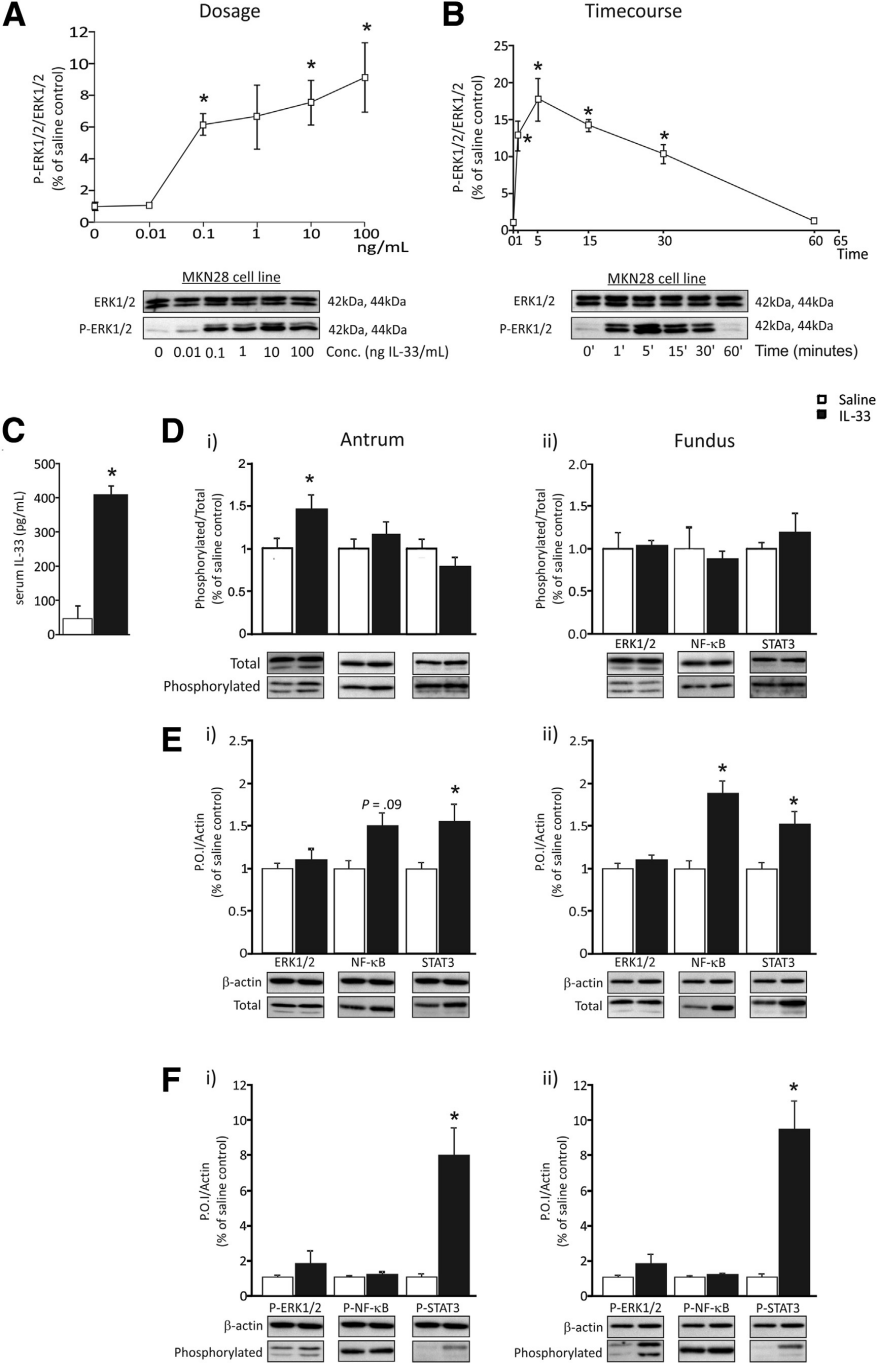
**The gastric mucosa is responsive to IL33 both in vitro and in vivo.** (*A*) Dose-dependent responsiveness of MKN28 cells to IL33 assessed through analysis of ERK1/2 activation (N = 3) (repeated twice). (*B*) Time-dependent responsiveness of MKN28 cells to IL33 assessed through analysis of ERK1/2 activation (N = 3) (repeated twice). (*C*) IL33 protein levels in sera of mice treated with IL33 for 7 days measured by ELISA. (*D*) Phosphorylation of ERK1/2, NF-κB, and STAT3 relative to total protein in the antrum and fundus of mice 24 hours after IL33 administration. (*E*) Amount of total protein of ERK1/2, NF-κB, and STAT3 relative to the housekeeping protein β-actin in the antrum and fundus of mice treated with IL33 for 7 days. (*F*) Amount of phosphorylation protein of ERK1/2, NF-κB, and STAT3 relative to the housekeeping protein β-actin in the antrum and fundus of mice treated with IL33 for 7 days. Saline, n = 10; IL33, n = 9; experimented was repeated 3 times. ∗Statistically significant, *P* ≤ .05. P.O.I., protein of interest.

To determine whether reduced IL33 expression during chronic *H pylori*–SS1 infection was caused by loss of SMCs we performed IL33 and UEA1 immunofluorescence on the stomach of 7-day and 2-month *H pylori*–SS1–infected mice (Figure 3*C*). *H pylori* infection (2 months), if anything, caused a slight increase in the amount of UEA1 staining, suggesting that at this time point *H pylori* infection increased SMCs and therefore reduced IL33 expression is independent of this process. In support of this finding mRNA expression of Muc5AC and TFF2 also was not affected by *H pylori* infection (Figure 2*Di*). These data suggest that *H pylori*–SS1 may act directly to suppress IL33 expression.

IL33 recently was reported to induce activation of ILC2,^4^ therefore we assessed whether altered IL33 mRNA expression during *H pylori*–SS1 was sufficient to alter the expression of the ILC2 genes, RAR-related orphan receptor alpha (RORa), amphiregulin, IL13, and GATA3 (Figure 2*E*). The mRNA expression of RORα and amphiregulin was not changed after 1 or 7 days of *H pylori*–SS1 infection (Figure 2*Ei* and *ii*). However, both were decreased after 2 months of *H pylori*–SS1 infection (Figure 2*Ei* and *ii*). In contrast, IL13 was not changed (Figure 2*Ei*), and neither was GATA3 (data not shown). To assess the specificity of these gene changes RORα, amphiregulin, IL13, and GATA3 also were assessed in the spleen after *H pylori*–SS1 infection. There was no difference in the mRNA expression of these genes at any time point tested (1 day, 7 days, 2 months, and point tested data not shown).

### Gastric Epithelial Cells Are Responsive to IL33 In Vitro

To determine the nature of the downstream signaling pathways activated in the stomach in response to IL33 treatment, a dose response and time course of signal activation in the human gastric cancer cell MKN28 was performed for up to 60 minutes. Activation pathways culminating in phosphorylation of NF-κB, ERK1/2, Akt, and STAT3 were evaluated. IL33 treatment did not alter the total expression or phosphorylation of these pathway mediators (data not shown), apart from ERK1/2. In dose-response experiments, MKN28 cells showed a progressive increase in pERK1/2, with increasing IL33 concentration (Figure 3*A*). Subsequently, MKN28 cells were treated with 100 ng IL33/mL in time course studies, and cells were harvested at time points from 0 to 60 minutes. MKN28 cells showed a peak of phosphorylated EKR1/2 after 5 minutes of IL33 treatment, followed by a rapid decrease to near-basal levels by 60 minutes (Figure 3*B*). These data show that the gastric cell line MKN28 is responsive to acute IL33 and that signaling is dependent predominantly on phosphorylated ERK1/2.

### Acute Administration of IL33 In Vivo Selectively Activates ERK1/2, and Chronic IL33 Activates STAT3 in the Gastric Mucosa

To understand the responsiveness of the whole stomach to IL33, mice were treated systemically with 1 μg of recombinant mouse IL33 daily by intraperitoneal injection, for either 24 hours (acute) or 7 days (chronic). Quantification of IL33 protein by ELISA in the serum of mice treated for 7 days, showed a 9-fold increase in circulating IL33 compared with basal levels in saline controls (Figure 3*C*). Acute administration of IL33 increased phosphorylated ERK1/2 to total ERK1/2 ratio by 46% in the antrum, whereas NF-κB and STAT3 were unchanged (Figure 3*D*). No changes in signaling were observed in the fundus (Figure 3*D*). After chronic IL33 treatment, the amount of total ERK1/2 to β-actin ratio was unchanged in the fundus or antrum (Figure 3*E*). However, there was an increase in the ratio of total NF-κB to β-actin as well as STAT3 to β-actin at this time point) (Figure 3*E*). Relative to the housekeeping protein, β-actin, phosphorylated ERK1/2, and phosphorylated NF-κB were unchanged in both the antrum and fundus (Figure 3*F*). In contrast, the ratio of phosphorylated STAT3/β-actin was increased substantially in both the antrum and the fundus (Figure 3*F*). These data suggest that IL33-mediated signaling pathways differ temporally in the gastric mucosa, with phosphorylated ERK1/2 predominating in acute settings and phosphorylated STAT3 induced chronically. In this context, hyperactivated STAT3 has been shown to be associated with progressive gastric inflammation and metaplasia.^18^

### Chronic IL33 Application Causes Inflammation, Atypical Metaplasia, and Hypoproliferation of the Gastric Mucosa

**Figure 4:**
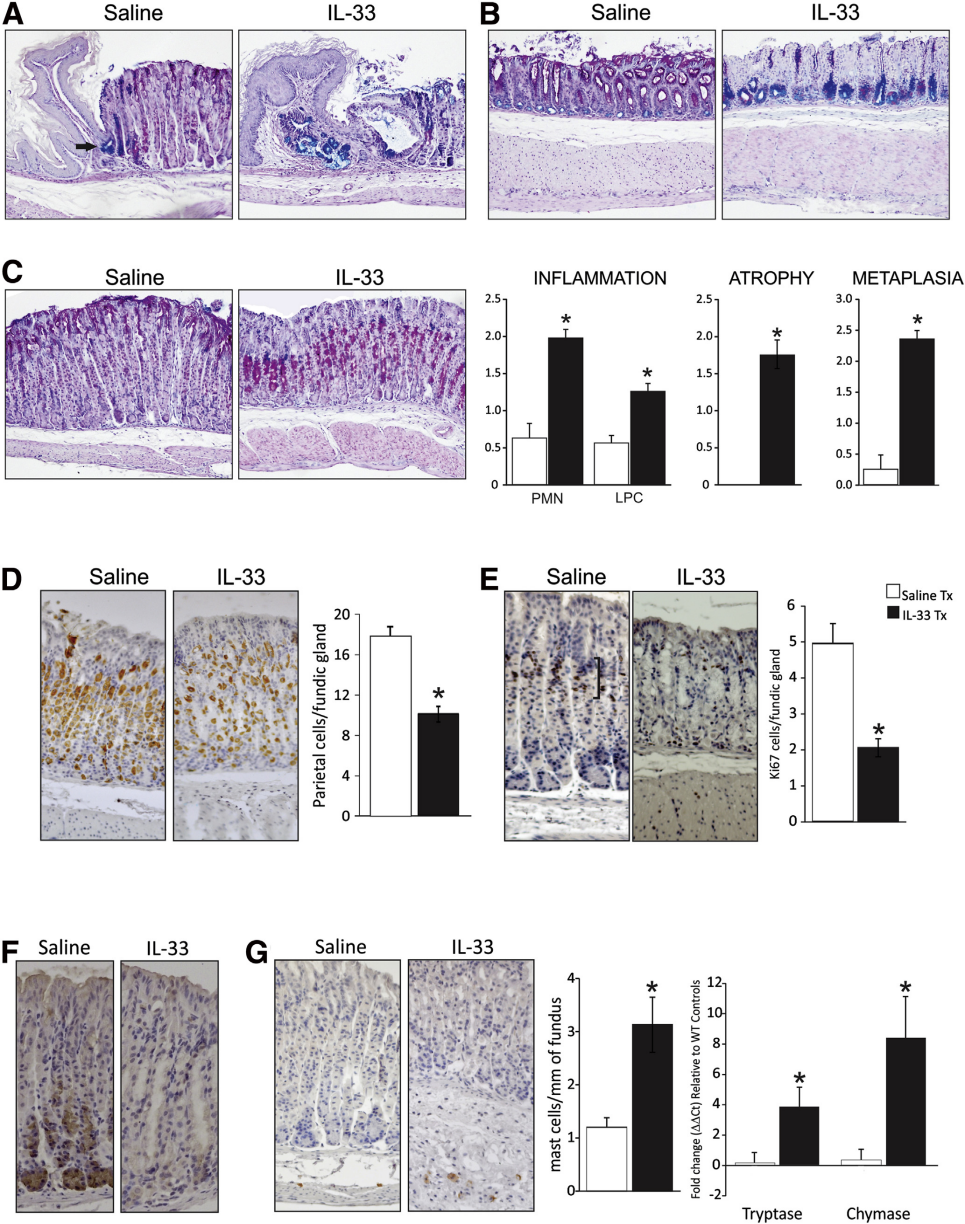
**IL33 treatment causes atypical gastric pathology.** (*A*) AB-PAS comparison of the cardiac region of the stomach between saline- and IL33-treated mice. (*B*) AB-PAS comparison of the antrum between saline- and IL33-treated mice. (*C*) AB-PAS comparison of the fundus between saline- and IL33-treated mice, and semiquantitative assessment of gastric inflammation, atrophy, and metaplasia. (*D*) Assessment of parietal cell atrophy with immunohistochemistry using antibody directed to H^+^/K^+^ adenosine triphosphatase β subunit. (*E*) Assessment of proliferation in the fundic glands with immunohistochemistry using antibody directed to Ki-67. (*F*) Assessment of chief cell atrophy with immunohistochemistry using antibody directed to intrinsic factor. (*G*) Assessment of mast cell infiltrate in the fundus with immunohistochemistry using antibody directed to mast cell chymase and mRNA expression of mast cell markers in IL33-treated mice relative to saline-treated mice and standardized to the housekeeper L32. Saline, n = 10; IL33, n = 9; experiment was repeated 3 times. ∗Statistically significant, *P* ≤ .05. Tx, treatment.

The stomach can be separated into 3 distinct gland types and/or regions according to the composition of gastric epithelial cells residing within each area: (1) the cardiac glands at the gastroesophageal junction, (2) the acid-secreting fundic glands that encompass the central portion of the stomach, and (3) the mucus-secreting antral glands that are most distal. Chronic IL33 treatment induced histopathologic changes in all 3 regions (Figure 4*A–C*). In a normal mouse stomach the cardiac glands are restricted to a very discrete group of Alcian blue (AB)–stained cells, indicative of acidic mucin secretion (Figure 4*A*, arrow). After IL33 treatment, the cardiac glands became disorganized, with an increased proportion of acidic mucin-producing cells in this region. This morphology was associated commonly with cystic dilatation in the adjacent fundus (Figure 4*A*). AB–periodic acid–Schiff (PAS) staining in the antrum showed a near-total loss of PAS-positive (neutral mucins) staining in IL33-dosed stomach sections in the surface and pit cells, and an increase in acidic mucins in cells at the base of the glands (Figure 4*B*). In the fundus, AB-PAS staining of IL33-treated stomachs highlighted atrophy, necrosis, metaplasia, inflammation, and thickening of the muscularis, with a distinct metaplasia characterized by an expanded population of PAS-expressing cells in the midregion of the glands, and a loss of mucin staining in SMCs (Figure 4*C*).

To better characterize the pathology observed in the stomach after chronic IL33 treatment, morphologic indices of inflammation, atrophy, and metaplasia were measured semiquantitatively. Assessment of immune infiltrate showed an increase in polymorphonuclear and lymphoplasmocytic infiltrate in the fundus, which was accompanied by atrophy and mucous metaplasia (Figure 4*C*). Atrophy was confirmed by H^+^K^+^ adenosine triphosphatase β staining, which showed a reduction in the number of parietal cells per fundic gland of IL33-treated mice (Figure 4*D*). Ki-67 staining showed a reduced number of proliferating cells in fundic glands (Figure 4*E*). In addition to this, a redistribution of Ki-67–positive cells from the neck region (Figure 4*E*, brackets) to the base of the glands and in the muscularis of IL33-treated mice was observed (Figure 4*E*, arrow). However, these Ki-67–positive cells appear to be infiltrating immune cells and not epithelial cells. To further define IL33-induced gastric pathology we performed immunohistochemistry for intrinsic factor, a marker of chief cells in mice, on 7-day IL33-treated mice (Figure 4*F*). In saline-treated mice there was obvious intrinsic factor staining of chief cells at the base of the gland, however, this staining was lost after IL33 treatment, reinforcing the observation that systemic administration of IL33 can cause gastric atrophy (Figure 4*F*).

In IL33-treated mice, mast cell infiltrate, defined by staining for mast cell chymase, was increased in the fundic mucosa (Figure 4*F*). In addition, mRNA expression of the mast cell markers, chymase and tryptase, were increased in the fundus of chronic IL33-treated mice (Figure 4*F*). There was no change in cell apoptosis as assessed by staining for activated caspase-3 (data not shown). These data show that prolonged exposure to IL33 has selective and profound effects on the gastric mucosa, including stasis of proliferation, loss of neutral mucin production in surface and pit cells, mucin metaplasia in antral and fundic glands, and an increase in inflammatory cells including mast cells. This emphasizes the potential key regulatory activity of IL33 in the stomach.

### Chronic IL33 Administration Promotes Myeloid Cell Accumulation in the Spleen

**Figure 5:**
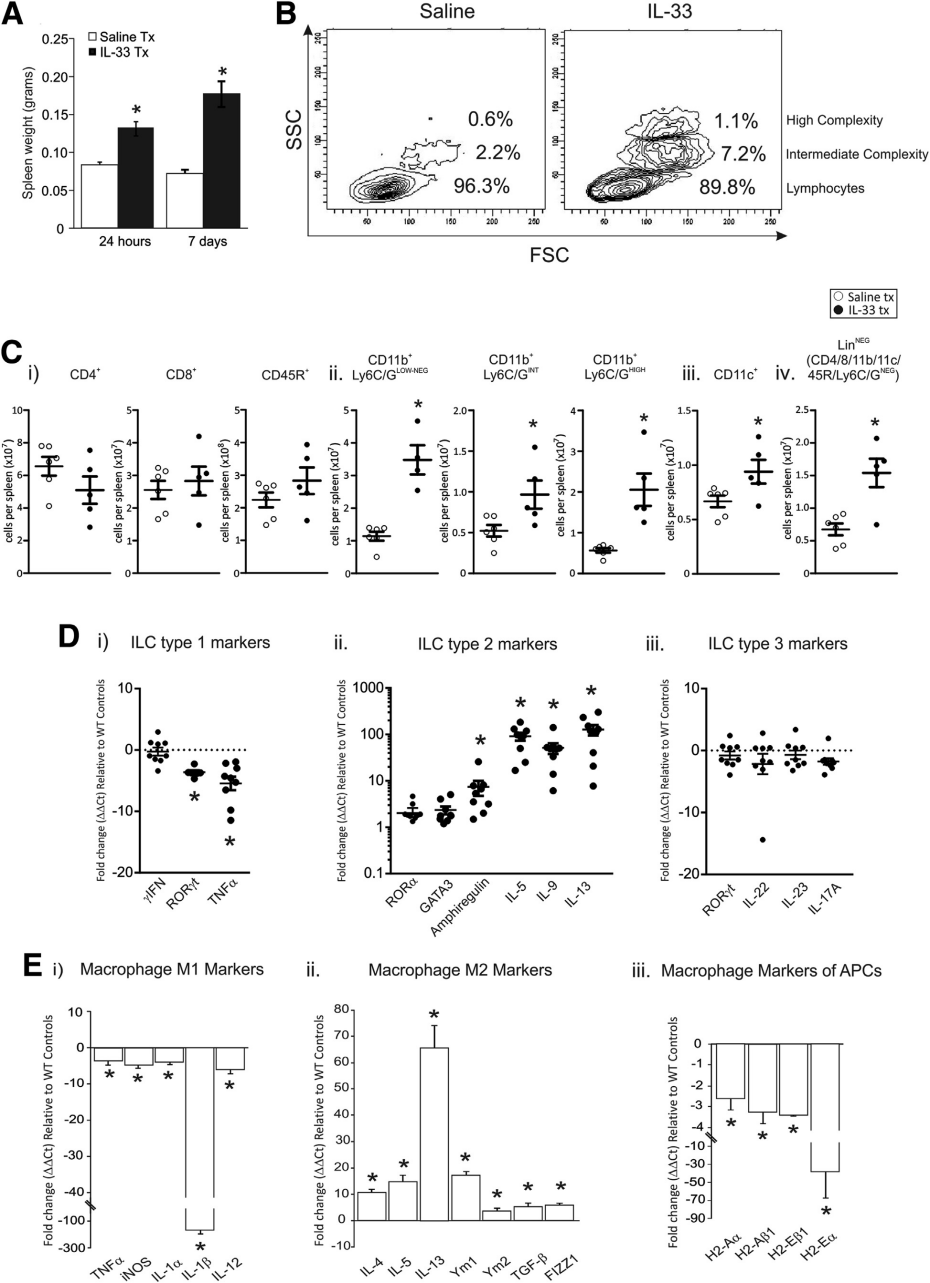
**Effects of IL33 on the spleen and peritoneal macrophages.** (*A*) Spleen weight after 24 hours and 7 days of IL33 treatment. (*B*) Size and complexity of immune cells in the spleen after 7 days of IL33 treatment compared with saline controls. (*C*) Total number of immunocyte types in the spleens of IL33-treated mice compared with saline controls: (*i*) CD4^+^, CD8^+^, and CD45R^+^; (*ii*) CD11b^+^Ly6C/G^LOW-NEG^, CD11b^+^Ly6C/G^INT^, and CD11b^+^Ly6C/G^HIGH^; (*iii*) CD11c^+^; and (*iv*) lineage-negative immunocytes (saline, n = 6; IL33, n = 5). (*D*) mRNA expression of markers of ILC in the spleen of 7-day IL33-treated mice all expressed relative to saline-treated mice and standardized to the housekeeper L32: (*i*) group 1 ILC markers; (*ii*) group 2 ILC markers; and (*iii*) group 3 ILC markers. (*E*) Assessment of peritoneal macrophage mRNA expression profile after IL33 treatment all expressed relative to saline-treated mice and standardized to the housekeeper L32: (*i*) M1 markers, (*ii*) M2 markers, (*iii*) markers of antigen-presenting cells (APCs). Saline, n = 10; IL33, n = 9; experiment was repeated twice. ∗Statistically significant, *P* ≤ .05. FSC, forward scatter; iNOS, inducible nitric oxide synthase; SSC, side scatter; Tx, treatment.

Initially, we measured the effects of systemic administration of IL33 on the peripheral immune system through analysis of the effects of IL33 on the spleen. IL33 is known to cause splenomegaly.^3^ Here, we expand on the mechanism by which IL33 can cause splenomegaly and the implication of this on gastric immune responses. After acute and chronic administration of IL33 we observed an increase in spleen weight of 50% after 24 hours and 120% after 7 days (Figure 5*A*). Flow cytometry was used to assess the effects of IL33 treatment on immune cell populations. The leukocytes were separated into 3 clear populations on the basis of their morphology: small size and low complexity (granularity) being predominantly lymphocytes, intermediate size and complexity, and high size and complexity. The last 2 populations are indicative of either myeloid cells or increased cellular activation. After IL33 treatment the spleen showed a decrease in the proportion of cells in the lymphocyte population and an increase in the proportion of larger and more highly complex cell populations (Figure 5*B*). In IL33-treated mice the total number of splenic CD4^+^ helper T cells, CD8^+^ cytotoxic T cells, and CD45R^+^ B cells were not changed compared with saline controls (Figure 5*Ci*). Conversely, the total number of splenic CD11b^+^Ly6C/G^NEG-LOW^ macrophages, CD11b^+^Ly6C/G^INT^ myeloid derived suppressor cells (MDSCs), CD11b^+^Ly6C/G^HIGH^ neutrophils, and CD11c^+^ dendritic cells (DCs) all were increased significantly (Figure 5*Cii* and *iii*). Lineage-negative cells (CD4/8/11b/11c/45R/Ly6C/G^NEG^), which include ILCs, also were quantified; and the total number of lineage-negative cells were increased in the spleen of IL33-treated mice (Figure 5*Civ*). In summary, IL33 administration causes an increase in the total number of splenic macrophages, neutrophils, and DCs, as well as lineage-negative cells, whereas T- and B-cell populations were not expanded.

We quantified ILC activation by measuring characteristic marker genes^23^ in the spleen after chronic administration of IL33 (Figure 5*Di–iii*). After chronic IL33 administration the ILC1 markers interferon-γ and tumor necrosis factor-α were decreased, whereas RORγt, a marker of ILC1 and ILC3, was not changed (Figure 5*Di*). In contrast, all ILC2 markers were increased in IL33-treated spleens compared with saline controls, including amphiregulin, IL5, IL9, and IL13 (Figure 5*Dii*), whereas RORα and GATA3 trended to increase but failed to reach statistical significance. Similar to ILC1, no differences were observed in the ILC3 markers RORγ, IL22, IL23, and IL17A (Figure 5*Diii*). These data show the ability of IL33 to specifically activate ILC2 to promote a Th2-biased response in the spleen.

### IL33 Polarizes Macrophages to the Alternatively Activated M2 Class

Macrophages are pivotal immune cells that participate in both the innate and adaptive immune response. In the lung, IL33 activates M2 macrophages leading to fibrosis,^20^ in addition there is precedent for macrophages playing an important role in the pathologic response of the gastric mucosa.^21^ Certain classes of macrophages have been shown to accelerate tumor progression^22^ and it now is accepted that, similar to T cells, a combination of effector signals can promote a macrophage to become polarized into either M1 or (alternatively activated) M2 class, differentiated by expression of specific Th1 or Th2 inflammatory markers.^23^ Therefore, peritoneal macrophages from chronic IL33-treated mice were isolated and M1 and M2 markers were quantified.

Overall, mRNA expression of M1-associated genes were decreased uniformly including tumor necrosis factor, inducible nitric oxide synthase, IL1α, IL1β, and IL12 (Figure 5*Ei*). In contrast, the expression of M2 genes was increased including IL4, IL5, and IL13 (Figure 5*Eii*). Additional M2 genes also were increased in IL33-treated macrophages including Ym1, Ym2, transforming growth factor-β, and FIZZ1 (Figure 5*Eii*). A second distinguishing feature of macrophages is their ability to present antigens through the major histocompatibility complex. Major histocompatibility complex genes H2-Aα, H2-aβ1, H2-Eα, and H2-Eβ1 all were decreased in IL33-treated macrophages (Figure 5*Eiii*). These data suggest that IL33-exposed peritoneal macrophages are polarized to an M2 phenotype and have a reduced capacity to present antigens.

### IL33 Promotes Expansion of Myeloid-Derived Immunocytes in the Stomach

**Figure 6:**
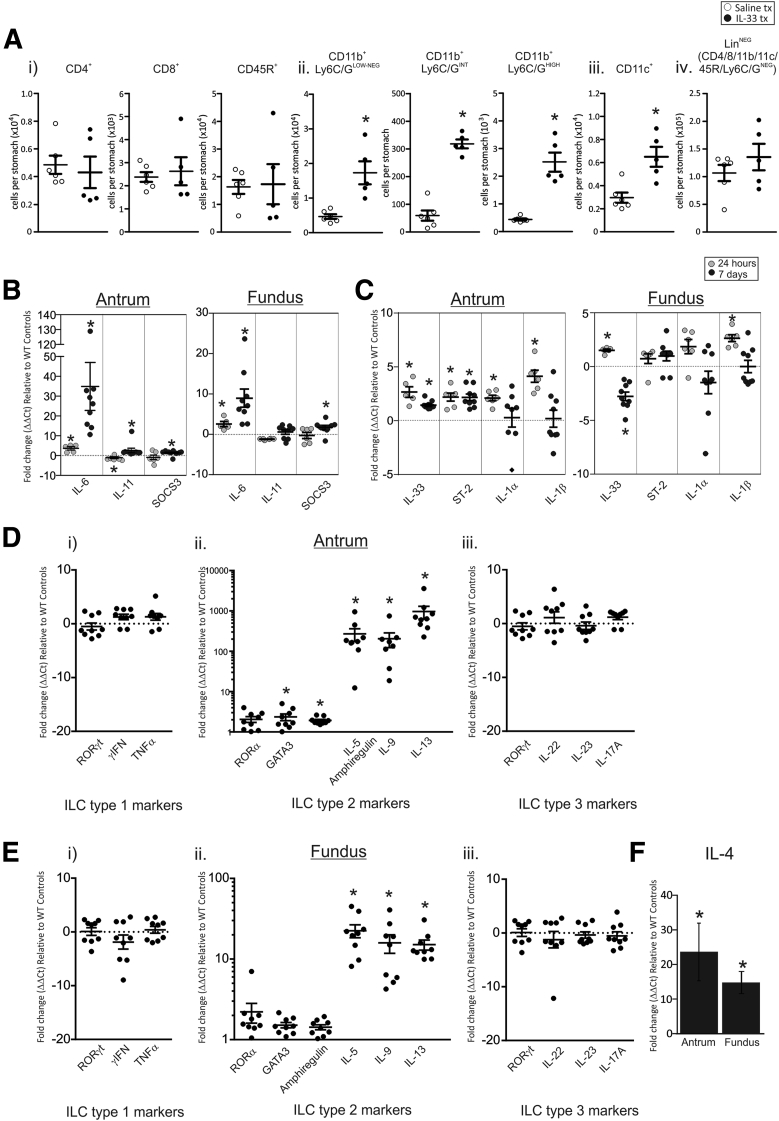
**Immunocyte populations in the stomach of 7-day IL33-treated mice and mRNA expression profile of ligands and regulators of gp130, IL1 cytokines, and ILC markers in the gastric mucosa of 24-hour and 7-day IL33-treated mice.** (*A*) Total number of immunocyte types in the stomach of IL33-treated mice compared with saline controls: (*i*) CD4^+^, CD8^+^, and CD45R^+^; (*ii*) CD11b^+^Ly6C/G^LOW-NEG^, CD11b^+^Ly6C/G^INT^, and CD11b^+^Ly6C/G^HIGH^; (*iii*) CD11c^+^; and (*iv*) lineage-negative immunocytes (saline, n = 6; IL33, n = 5). (*B*) mRNA expression of gp130 ligands in the antrum and fundus of 24-hour and 7-day IL33-treated mice all expressed relative to saline-treated mice and standardized to the housekeeper L32. (*C*) mRNA expression of IL1 family cytokines in the antrum and fundus of 24-hour and 7-day IL33-treated mice. (*D*) Antrum and (*E*) fundus, expression of ILC markers: (*i*) group 1 ILC markers; (*ii*) group 2 ILC markers; and (*iii*) group 3 ILC markers. (*E*) IL4 expression in the antrum and fundus of 7-day IL33-treated mice. All values are expressed relative to saline-treated mice and standardized to the housekeeper L32. Saline, n = 10; IL33, n = 9; experiment repeated twice. ∗Statistically significant, *P* ≤ .05.

We previously showed that IL33 promotes expansion of myeloid-derived immunocytes in the spleen (Figure 5*B* and *C*), therefore we wanted to determine whether immunocyte populations were altered similarly in the stomach. Stomach immunocyte populations were assessed using fluorescence-activated cell sorter analysis and the total cell number per stomach is shown in Figure 6*A*. Similar to the spleen, IL33-treated stomachs showed no differential in the number of CD4^+^ helper T cells, CD8^+^ cytotoxic T cells, and CD45R^+^ B cells (Figure 6*Ai*). Consistent with the spleen there was an increase in the total number of stomach CD11b^+^Ly6C/G^NEG-LOW^ macrophages, CD11b^+^Ly6C/G^INT^ MDSCs, CD11b^+^Ly6C/G^HIGH^ neutrophils, and CD11c^+^ DCs (Figure 6*Aii*), however, we observed no expansion of lineage-negative cells (Figure 6*Aiv*). These data show that IL33 promotes the expansion of myeloid-derived immunocytes in the stomach.

### IL6 May Induce STAT3 Activation After Chronic Administration of IL33

IL33 does not directly promote STAT3 activation in a human gastric cell line (Figure 3*A* and *B*) or in the stomach after acute administration; however, STAT3 is activated in vivo after 7 days of IL33 administration (Figure 3*D–F*). To determine the identity of the indirect activator of STAT3 in IL33-treated stomachs, we quantified the 2 main gastric gp130/STAT3 ligands, IL6 and IL11, as well as the negative regulator of STAT3 signaling, suppressor of cytokine signaling 3. Acute administration of IL33 caused IL6 mRNA to be increased in both the antrum and fundus by 2- to 4-fold (Figure 6*B*), whereas IL11 mRNA was decreased throughout the stomach (Figure 6*B*). After 7 days of IL33 treatment, IL6 preferentially was activated (Figure 6*B*). These data show that IL6 expression is induced downstream of both acute and chronic IL33 administration, and may be a key activator of STAT3 after IL33 treatment.

### IL33 Administration Enhances Activity of ILC2 Cells in the Stomach

mRNA expression of the IL1 family cytokines IL1α, IL1β, IL33, and its receptor ST2 were measured to assess the inflammatory response in the stomach after exogenous IL33 treatment. In the antrum of acute IL33-treated mice there was an increase in IL33, ST2, IL1α, and IL1β (Figure 6*C*), however, this response was suppressed with chronic treatment, with IL33 and ST2 only marginally increased and IL1α and IL1β unchanged (Figure 6*C*). A similar outcome was observed for the fundus (Figure 6*C*).

IL33 promotes the expansion of myeloid-derived cells in the stomach (Figure 6*A*), however, it previously was reported that IL33 can activate ILC2 and Th2 immunocytes, and thus initiate a Th2 cytokine response.^4^ Therefore, to assess whether IL33 could activate ILC2 and Th2 immunocytes in the stomach (Figure 6*A*), we quantified the mRNA expression of types 1, 2, and 3 ILCs in the stomachs of mice treated with IL33 for 7 days. A very similar response was observed in both the antrum and fundus, although the magnitude of the response in the former with respect to ILC2 markers was 10-fold greater than in the latter. In both mucosae, markers for ILC1 and ILC3 were unchanged in response to IL33 (Figure 6*D*), however, the ILC2 markers IL5, IL9, and IL13 all were increased greatly in their mRNA expression (antrum 200- to 1000-fold, Figure 6*D*; and fundus 15- to 23-fold, Figure 6*E*), whereas RORα, GATA3, and amphiregulin were increased only marginally (Figure 6*D* and *E*). In addition, the Th2 cytokine, IL4, also strongly was up-regulated in both antrum and fundus (13- to 26-fold, Figure 6*F*) co-incident with the strong gastric Th2 response to IL33. These findings show IL33 to be multifunctional in influencing the gastric immune response, and that prolonged exposure to IL33 results in a Th2-biased response indicative of Th2 lymphoid-derived cell and ILC2 activation, despite these immunocyte populations not being expanded in the stomach (Figure 6*A*). Furthermore, IL9 can induce STAT3 phosphorylation,^24^, ^25^ and therefore, along with IL6, may contribute to the increase in phosphorylated STAT3 observed in these mice.

### IL33-Induced Gastric Pathology Is Dependent on Mature Lymphoid-Derived Cells

**Figure 7:**
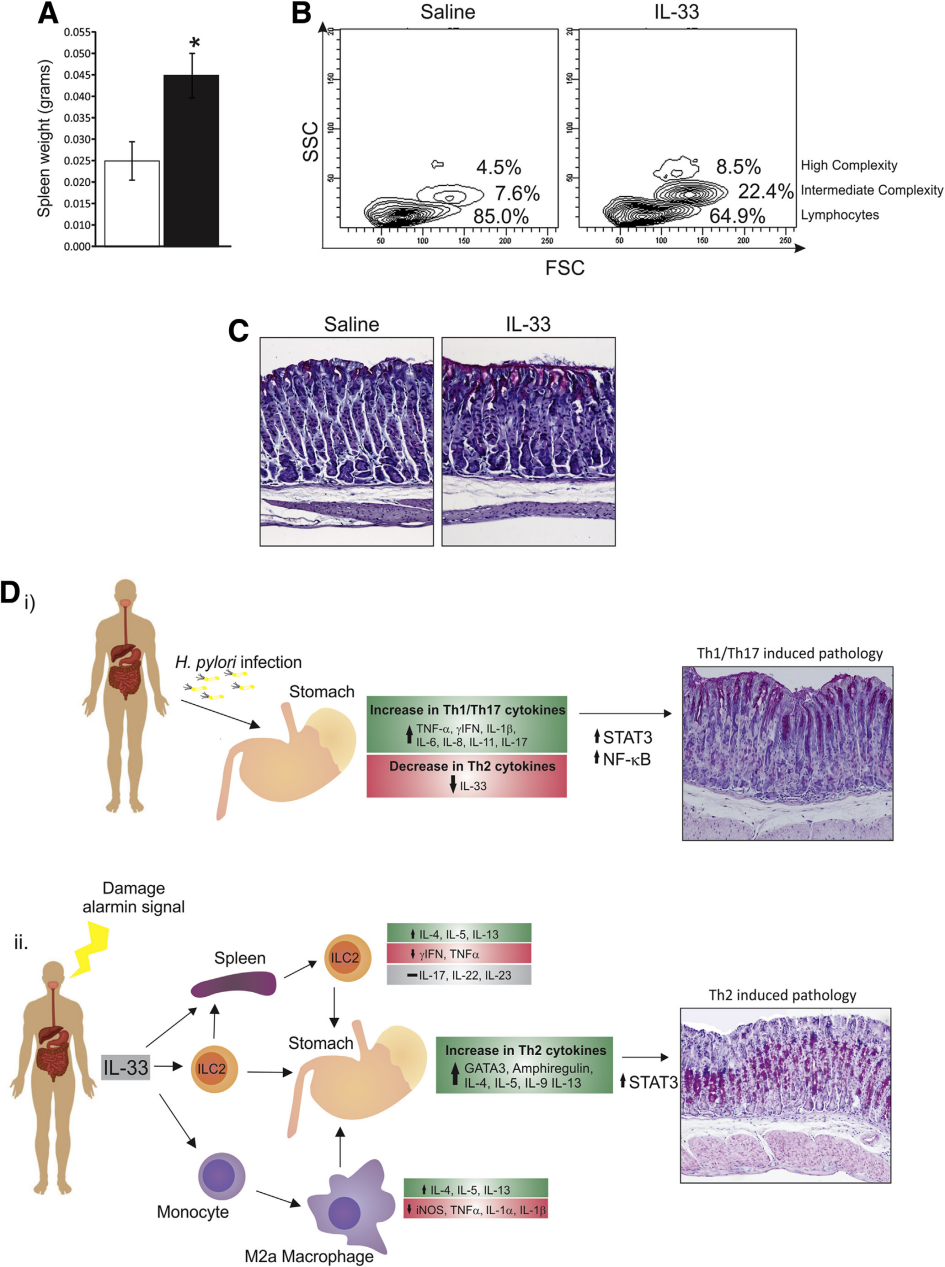
**Effects of IL33 on the spleen and stomach of lymphocyte-deficient mice.** (*A*) Spleen weight of IL33-treated Rag-1^-/-^ mice. (*B*) Size and complexity of immune cells in the spleen of 7-day IL33-treated Rag-1^-/-^ mice. (*C*) AB-PAS staining of the fundus of Rag-1^-/-^ mice. WT-saline, n = 6; WT–IL33, n = 6; Rag-1^-/-^–saline, n = 6; Rag-1^-/-^–IL33, n = 6. (*D*) Summary of immunologic changes and gastric pathology after (*i*) *H pylor*i infection or (*ii*) 7 days of IL33 treatment. ∗Statistically significant, *P* ≤ .05. SSC, standard saline citrate.

After chronic IL33 treatment, WT mice develop gastric metaplasia and hypoplasia with characteristic loss of surface mucins and glandular mucous metaplasia. IL33 gastropathy is associated with marked activation of Th2 lymphocytes and ILC2 and activation and expansion M2 macrophage recruitment, shown by fluorescence-activated cell sorter analysis and mRNA expression profiles. To clarify which immunocyte population promotes this mucosal response, Rag-1^-/-^ mice, which lack mature lymphocytes including B, T, and natural killer T cells,^26^ but develop normal myeloid and ILC lineages, were treated with IL33 or saline for 7 days by intraperitoneal injection. IL33-treated Rag-1^-/-^ mice had a doubling of spleen weight compared with littermate controls, consistent with findings in WT mice (Figure 7*A*). They also showed a decrease in lymphocyte populations and an increase in intermediate and high complex cell populations (Figure 7*B*). These cell population changes were similar to those of IL33-treated WT mice (Figure 5*B*). Despite this, Rag-1^-/-^ mice showed no evidence of gastric metaplasia or infiltration of immunocytes after IL33 treatment, suggesting that lymphocytes are required for IL33-induced gastric pathology (Figure 7*C*). In WT mice, STAT3 activation coincided with high IL6 and IL9 mRNA expression. IL6, and particularly IL9, are produced by T cells; therefore, it is most likely that T cells drive IL33-mediated Th2/STAT3 gastric pathology.

## Discussion

IL33 now is recognized as an alarmin, capable of responding to adverse circumstances such as infection or mucosal damage in the lung and colon, and can promote Th2 immunity.^2^ Similar to the lung and colon, the stomach epithelium requires a stringent, balanced immune response to ensure homeostasis, and dysfunctional immunity can lead to disease progression, such as that after *H pylori* infection. We have shown that IL33 ideally is expressed in gastric pit mucous cells to modulate local immune responses. In addition to being localized to gastric pit cells, a small portion of IL33-positive cells were co-localized with Ki-67. This suggests that IL33 is expressed once progenitor cells begin to differentiate into presurface mucous cells and IL33 continues to be expressed by SMCs within gastric pits. However, as SMCs continue to mature and migrate toward the tips of the glands, IL33 is suppressed (Figure 2*C*). This loss of IL33 may be important in preventing inappropriate activation of gastric immunity. We also show that IL33 can be induced acutely in response to bacterial antigen challenge and epithelial damage. Collectively, the data show that IL33 is a stomach alarmin. In addition, IL33 expression is increased chronically when the environmental bacterial load is increased, suggesting that it plays an important role in maintaining correct luminal microbiota.

In keeping with an alarmin function, IL33 mRNA expression is increased acutely after *H pylori* infection in mice; however, its expression is decreased 2 months after infection. Similarly, IL33 is lost in human *H pylori*–positive biopsy specimens compared with negative biopsy specimens. Chronic *H pylori* infection exacerbates Th1/Th17 inflammation, at the expense of Th2 immunity, leading to disease progression (Figure 7*Di*). This skewed immunity during prolonged *H pylori* infection is well established as fundamental for gastric carcinoma development, suggesting that the loss of Th2 immunity contributes significantly to disease progression. IL33 recently was shown to modulate the function of regulatory T cells in the intestine.^27^ Regulatory T cells are potent immune suppressors and although their abundance in gastric tumors negatively correlates to patient survival, increased levels of the regulatory T-cell protein, FoxP3, a marker of regulatory T-cell activity, within gastric cancer cells coincides with a good prognosis.^28^ In addition, FoxP3 has been shown to reduce tumor growth.^28^ Therefore, IL33 may inhibit *H pylori*–induced gastric disease by enhancing regulatory T-cell function, but not expansion. Furthermore, under circumstances in which Th2 immunity is increased during *H pylori* infection, such as co-infection with gut parasites, resultant gastric pathology is reduced, or does not eventuate.^29^ In addition, some human populations with a high gut parasite burden but equivalent levels of *H pylori* infection have a reduced incidence of gastric cancer.^30^ This suggests that mechanisms that promote Th2 immunity can limit the development of *H pylori*–induced gastric pathology. Therefore, the inhibition of gastric IL33 in response to chronic *H pylori* infection may be a key event in gastric cancer progression by preventing induction of Th2 immunity, and skewing the local immune response to Th1/Th17 (Figure 7*Di*).

TFF2 expression is required for normal basal IL33 expression in the lung.^12^ Similar to the lung, loss of TFF2 causes a reduction in IL33 expression in the stomach, showing that IL33 is regulated at least partially by TFF2. Similar to IL33, TFF2 expression is lost during chronic *H pylori* infection in human beings, and its expression decreases progressively as cancer advances.^19^ Furthermore, TFF2 is crucial for maintaining gastric gland integrity and repair,^31^, ^32^ a function to which IL33 also may contribute, as shown by the release of IL33 at sites of injury after aspirin insult. In addition, TFF2 null mice spontaneously develop gastric pathology and are more susceptible to *H pylori* infection than WT mice.^33^ Pathologic outcomes in this mouse model may develop owing to reduced IL33 expression, particularly in the setting of *H pylori* infection, in which TFF2 null mice may have reduced IL33/Th2 immunity.

Interestingly, during *H pylori* infection, loss of IL33 expression does not correlate to a loss of SMCs or TFF2 expression, suggesting that *H pylori* may suppress IL33 directly. It also is possible that IL33 is regulated by additional gastric-associated genes besides TFF2. Considering that IL33 is expressed in SMCs, functions as an alarmin, and appears to regulate gastric homeostasis and wound healing, it is plausible to suggest that gastric homeostatic genes, necessary for normal gastric function, such as TFF1 and gastrokines 1 and 2, also may influence IL33 expression, and altered expression of these and other genes during chronic *H pylori* infection may cause a decrease in IL33 expression.

Although we show here that IL33 is decreased after chronic *H pylori* infection, it recently was reported that IL33 serum levels are increased during gastric cancer.^34^ Sun et al^34^ observed a positive correlation between IL33 serum levels and cancer severity, with individuals with the highest IL33 serum levels having the most aggressive cancers with metastatic properties. This suggests that IL33 serum levels may be a potential biomarker for gastric cancer. Although this is a highly interesting finding, it is worth noting that increased serum IL33 levels were typically in the setting of metastatic gastric cancer and it is not clear if the source of IL33 in this setting was gastric in origin. In contrast, here we clearly have shown the capacity of IL33 to act as a stomach alarmin and provide evidence to suggest that its suppression during *H pylori* infection may influence disease progression.

Acute and chronic IL33-treated mice present with increased spleen weight as previously reported.^35^ Acute administration was consistent with alarmin function; that is, activation of ERK1/2 in the gastric mucosa and increased expression of innate cytokines, IL1α, IL1β, and IL6 shows the capacity of IL33 to mediate gastric inflammation and act as a stomach alarmin. In contrast, chronic administration caused a distinctly different outcome. ERK1/2 was no longer activated, and expression of IL1α or IL1β was not increased. However, STAT3 was highly activated, leading to Th2-mediated gastric metaplasia, suggesting that IL33 may be capable of driving gastric disease under specific circumstances. In IL33-treated mice, the fundus was atrophic (Figure 7*Dii*), and the cardiac stomach was greatly expanded and metaplastic. Such cardiac stomach pathologies have been associated with progression to Barrett’s esophagus, a precursor of esophageal cancer.^36^ The incidence of Barrett’s esophagus is increasing steadily, coincident with a gradual decrease in *H pylori* infections and gastric cancer incidence.^37^, ^38^, ^39^ In addition, *H pylori* colonization also has been shown to protect against Barrett’s esophagus.^37^, ^38^, ^39^ Therefore, IL33 may promote cardiac pathology leading to Barrett’s esophagus, and *H pylori* may protect against Barrett’s esophagus by suppressing IL33/Th2 immunity. This highlights the importance of balanced gastric immunity. Although we suggest that the loss of IL33 expression during *H pylori* infection can promote *H pylori*–induced gastric pathology, we also show that excessive IL33/Th2 immunity can drive pathology. Furthermore, despite the immune response being vastly different (*H pylori* induces a Th1/Th17 biased response while IL33 induces a Th2 biased response), the outcome appears to be similar because preneoplastic changes occur in both settings (Figure 7*D*). In keeping with recent findings,^37^, ^38^, ^39^ these data suggest that *H pylori* may be a beneficial organism that under certain circumstances may help to ensure that gastric immunity is tightly regulated.

Flow cytometric analysis of spleen tissue showed that IL33-treated mice had a marked increase in neutrophils, MDSCs, DCs, and macrophages. In addition, IL33 administration caused activation of ILC2s in the spleen, as shown by the increase in size and complexity of lineage-negative cells and an increase in mRNA expression of ILC2- and Th2-associated genes. Neutrophils, MDSCs, DCs, and macrophages also substantially were increased in the stomach after IL33 treatment, as were ILC2- and Th2-associated genes. ILC2s are influential in modulating immunocytes during infection and lymphoid cell development by driving Th2 activation, macrophage polarization, and promoting B-cell production, and are necessary for T-cell development, highlighting their importance for mounting a successful Th2 immune response to infection.^40^, ^41^ Our data show that IL33 promotes ILC2 activation in both the spleen and stomach (summarized in Figure 7*D*), which coincides with morphologic changes in the gastric mucosa (Figure 7*Dii*), including the accumulation of mast cells, which previously were shown to associate with metaplasia in other tissues, particularly in the lung.^42^, ^43^ IL33 also caused the polarization of peritoneal macrophages to the M2 phenotype, expressing high levels of IL4, IL5, and IL13. M2 macrophage populations are recognized as tumor-associated macrophages and can drive carcinomas.^44^, ^45^ Therefore, by analogy, it is reasonable to suggest that IL33 may promote differentiation and accumulation in the stomach of M2 macrophages, which also contribute to IL33-induced gastric pathology.

Although gastric pathology appears to be dependent on STAT3 activation rather than NF-κB– or ERK1/2-mediated signaling, we have shown that IL33 does not activate STAT3 directly. This suggests that IL33-induced gastric pathology is dependent on infiltrating immune cells, which in turn induce STAT3, leading to gastropathy. In chronic IL33-treated WT mice, the established STAT3 inducers, IL6 and IL9, were highly up-regulated in the stomach. Although IL6 commonly is secreted by most immunocytes, recent work has suggested that IL9 is predominately a product of T cells.^42^, ^46^, ^47^ This suggests that T cells are most likely responsible for driving IL33-mediated gastric pathology through up-regulation of IL6 and IL9. In support of this, we show that lymphocytes are crucial in IL33-mediated gastric pathology as shown by the absence of gastric pathology in lymphocyte-deficient Rag1^-/-^ mice. Interestingly, the number of lymphocytes was not altered in the spleen and stomach of IL33-treated mice despite the predominant effects of IL33 on the spleen and stomach appearing to be Th2-immune driven and their necessity for gastric pathology.

Here, we show that IL33 is a stomach alarmin, which is induced acutely during chemical damage or infection, but is suppressed by chronic *H pylori* infection. Furthermore, we show that the local gastric homeostatic factor TFF2 positively regulates IL33 expression in the stomach because TFF2 null mice have substantially reduced IL33 expression. This loss of IL33 may be a pivotal event in skewing the immune response in chronic *H pylori*–induced gastric pathologic progression. Furthermore, we show the ability of IL33 to potently activate ILC2s and T cells in the gastric mucosa, highlighting its ability to initiate an effective Th2 immune response. Although an acute Th2 response may be beneficial by preventing unchecked Th1/Th17 inflammation, we have shown that chronic Th2 activation produces M2 macrophage polarization and increased STAT3 activation, both of which cause gastropathy,^20^, ^48^, ^49^ being particularly marked in the gastric cardia.

## Figures and Tables

**Supplementary Table 1:** Fluorochromes Used for Identification of Immune Cells by Flow Cytometry

Marker	Fluorochromes	Company	Reference no.
CD4	V450	BD Biosciences^a^	560468
CD8a	APC-Cy7	BD Biosciences^a^	561967
CD11b	PE	BD Biosciences^a^	557397
CD11c	APC	BD Biosciences^a^	550261
CD45R/B220	FITC	BD Biosciences^a^	553087
Ly-6G and Ly6C (Gr-1)	PerCP-Cy5.5	BD Biosciences^a^	552093


Immune cell	Fluorochromes
Th CD4+	V450+/APC-Cy7-
CTL CD8+	V450-/APC-Cy7+
CD45R/B220^+^ B cells	CD8a^-^/CD45R/B220^+^
CD11b^+^Ly6C/G^net-low^ macrophages	CD11b^+^/Ly-6G and Ly6C (Gr-1)
CD11b^+^Ly6C/G^+high^ neutrophils	CD11b^+^/Ly-6G and Ly6C (Gr-1)
CD11c^+^ DCs	CD4^+^/CD11c^+^, CD8a^+^/CD11c^+-^, CD4^-^/ CD8a^-^/CD11c^+^

a BD Biosciences, San Diego, CA.

**Supplementary Table 2:** Primer Sequences Used for mRNA Expression Analyses via QRT-PCR

Gene	Forward	Reverse
IL33	GGTGTGGATGGGAAGAAGCTG	GAGGACTTTTTGTGAAGGACG
ST2	CACCAGATCATTCACAGTTG	CCACTTCCATTGTGTGGTTGTA
IL1α	AACCCATGATCTGGAAGAGACC	TGGTGCTGAGATAGTGTTTGTCC
IL1β	CAGGCAGTATCACTCATTGTGG	GTGCAGTTGTCTAATGGGAACG
IL4	GTCATCCTGCTCTTCTTTCTCG	TCACTCTCTGTGGTTCTTCG
IL5	TTGACAAGCAATGAGACGATGAG	GCCCCTGAAAGATTTCTCCAA
IL6	ACAAAGCCAGAGTCCTTCAGAGA	CTGTTAGGAGAGCATTGGAAATTG
IL9	CGTCCCAACTGATGATTGTACC	TGCATCTCTGTCTTCTGGAACG
IL11	CTGCAAGCCCGACTGGAA	AGGCCAGGCGAGACATCA
IL12	TCTAGATGCTGGCCAGTACACC	CCAAATGTGCTTGTCACCAC
IL13	GTGCCAAGATCTGTGTCTCTCC	TTACAGAGGCCATGCAATATCC
IL17A	TCTGTGTCTCTGATGCTGTTGC	ACATTCTGGAGGAAGTCCTTGG
IL22	TCGTCAACCGCACCTTTATG	GCCGGACATCTGTGTTGTTATC
IL23	GAGCAACTTCACACCTCCCTACTAG	GGCCTGGAGGCTTCGAA
TNF-α	TATGTCTCAGCCTCTTCTCATTCC	ATGATCTGAGTGTGAGGGTCTGG
IFN-γ	TCAAGTGGCATAGATGTGGAAGAA	TGGCTCTGCAGGATTTTCATG
TGF-β	AATACAGGGCTTTCGATTCAGC	TAGTTGGTATCCAGGGCTCTCC
iNOS	CCCTCCAGTGTCTGGGAGCA	TGCTTGTCACCACCAGCAGT
Ym1	TCTGGTGAAGGAAATGCGTAAA	GCAGCCTTGGAATGTCTTTCTC
Ym2	TGCCCCGTTCAGTTCCAT	TTGACGAAGGAATCTGATAACTGACT
Fizz1	CCTGCTGGGATGACTGCTACT	CTCCACTCTGGATCTCCCAAGAT
RORγt	GAGTTTGCCAAGCGGCTTT	TCCATTGCTCCTGCTTTCAGT
RORα	GCGGTTGACCTCGGCATAT	ACGCTGGACTCTGCTGTTACC
GATA3	CGAGACATAGAGAGCTACGCAATC	ACCTGAGTAGCAAGGAGCGTAGA
Amphiregulin	CTATCTTTGTCTGCCATCATC	CAACTTTTACCCTTGCAATTGCC
H2-Aα	CCAGGGCCTTTATGAGTCACA	GTCACCCAGCACACCACTTCT
H2-Aβ1	GGAGACTCCGAAAGGCATTTC	TGCGTCCCGTTGGTGAA
H2-Eα	GCGGAGTTCTATCTTTTACCAGACA	CTCATCGCCGTCAAAGTCAA
H2-Eβ1	TGTGTGGCAGCTGTGATCCT	AAACCATGGTCTGGAGTCTCTGA
SOCS3	AATACCTTTGACAAGCGGAC	TGCTACTGCCTCTTCACAAC
Chymase	TCTTACTCTTCATCTGCTGCTCCTT	CCTCCAATGATCTCTCCAGCTT
Tryptase	TGTGTGGGACCGGATGTTG	TGGTCATGGTAATAGAGGTACTGCTT

IFN, interferon; iNOS, inducible nitric oxide synthase; SOCS3, suppressor of cytokine signaling 3; TGF, transforming growth factor; TNF, tumor necrosis factor.

**Supplementary Table 3:** Antibodies Used for Immunoblotting, Immunohistochemistry, and Immunofluorescence

Specificity	Antibody	Manufacturer	Reference no.
IL33	IL33 antibody	R&D Systems^a^	AF3626
β-actin	β-actin (13E5)	Cell Signaling^b^	4970
Stat3	STAT3	Cell Signaling^b^	9132
p-stat3	Phospho-STAT3 (Tyr705)	Cell Signaling^b^	9131
ERK1/2	P44/42 mitogen-activated protein kinase	Cell Signaling^b^	9102
p-ERK1/2	Phospho-p44/42 mitogen-activated protein kinase (Thr202/Tyr204)	Cell Signaling^b^	4377
H/K ATPase-β	β subunit of H/K ATPase (Dr Ian VanDriel)	Bio21 Institute^c^	-
Ki-67	Ki-67 proliferation marker	Abcam^d^	Ab15580
Chymase	mast cell chymase	Abcam^d^	Ab111239
UEA1	*Ulex europaeus*	Sigma^e^	L8262
Muc5AC	Mucin 5AC	Abcam^d^	Ab3649
Ki-67	Ki-67 proliferation marker	Thermo Scientific^f^	RM-9106-SO
Intrinsic factor	Intrinsic factor (Dr David Alpers)	-	-

ATPase, adenosine triphosphatase.

a R&D Systems, Minneapolis, MN.

b Cell Signaling, Danvers, MA.

c Bio21 Institute, Parkville, Australia.

d Abcam, Cambridge, MA.

e Sigma, Cream Ridge, MA.

f Thermo Scientific, Waltham, MA.
